# The Role of NF-*κ*B in PPAR*α*-Mediated Hepatocarcinogenesis

**DOI:** 10.1155/2008/286249

**Published:** 2009-01-29

**Authors:** Howard P. Glauert, Karen Calfee-Mason, Yixin Li, Vani Nilakantan, Michelle L. Twaroski, Job Tharappel, Brett T. Spear

**Affiliations:** ^1^Graduate Center for Nutritional Sciences, University of Kentucky, Lexington, KY 40536, USA; ^2^College of Health and Human Services, Western Kentucky University, Bowling Green, KY 42101, USA; ^3^Applied Biosystems, Foster City, CA 94404, USA; ^4^Division of Transplant Surgery, Kidney Disease Center, Medical College of Wisconsin, Milwaukee, WI 53226, USA; ^5^Food and Drug Administration, CFSAN/OFAS/DFCN, College Park, MD 20740, USA; ^6^Department of Microbiology, Immunology, and Molecular Genetics, University of Kentucky, Lexington, KY 40536, USA

## Abstract

In this review, the role of NF-*κ*B in the induction of hepatocarcinogenesis by peroxisome proliferators is examined. The administration of peroxisome proliferators for more than a three-day period leads to the activation of NF-*κ*B in the livers of rats and mice. On the other hand, peroxisome proliferator activated receptor-*α* (PPAR*α*) activation in non-hepatic tissues can lead to the inhibition of NF-*κ*B activation. Several lines of evidence support the hypothesis that the activation of NF-*κ*B by peroxisome proliferators in the liver is mediated by oxidative stress. The role of NF-*κ*B in peroxisome proliferator-induced hepatocarcinogenesis has been examined using NF-*κ*B knockout models. Specifically, the induction of cell proliferation and the promotion of liver carcinogenesis are inhibited in mice lacking the p50 subunit of NF-*κ*B. Overall, the activation of NF-*κ*B appears to be important in the carcinogenic activity of peroxisome proliferators.

## 1. INTRODUCTION

Peroxisome proliferators (also known as PPAR*α* agonists) are a group of chemically distinct compounds capable of eliciting persistent peroxisome proliferation in hepatocytes and inducing liver tumors in rats and mice [1, 2]. These chemicals activate the peroxisome proliferator-activated receptor *α* (PPAR*α*) which is essential for the carcinogenic properties of these agents [3]. The administration of peroxisome proliferators increases the size and number of peroxisomes and activates genes encoding several enzymes of the peroxisomal *β*-oxidation pathway [4, 5]. The rate-limiting enzyme of this pathway, fatty acyl CoA oxidase (FAO), produces hydrogen peroxide (H_2_O_2_) as a by-product. In addition, the induction of cytochrome P450 4A enzymes by peroxisome proliferators, which is mediated through PPAR*α*, produces superoxide anions as by-products [6]. Oxidative stress may be important in the toxicity and carcinogenicity of peroxisome proliferators, via the induction of lipid peroxidation, oxidative DNA damage, and/or changes in gene expression [1, 7]. The changes in gene expression may be brought about in part by the activation of the transcription factor NF-*κ*B, which is known to be induced by oxidative stress. In this review, we will discuss the evidence that peroxisome proliferators activate NF-*κ*B, whether NF-*κ*B activation is necessary for the induction of carcinogenesis by peroxisome proliferators, and the mechanisms by which peroxisome proliferators may influence NF-*κ*B activation.

## 2. NF-*κ*B

Nuclear factor-*κ*B (NF-*κ*B) is a eukaryotic transcription factor family consisting of the following proteins: p50 (NF-*κ*B1), p65 (RelA), p52 (NF-*κ*B2), c-Rel, and RelB. It is normally found in the cytoplasm as an inactive dimer, with the most common being the p50–p65 heterodimer, bound to an inhibitory subunit, I*κ*B, which also has several family members, including I*κ*B*α*, I*κ*B*β*, I*κ*B*γ*, and I*κ*B*ε* [8]. Upon activation, NF-*κ*B is released from I*κ*B and translocates to the nucleus, where it binds target sequences of responsive genes. This process requires the phosphorylation of I*κ*B, followed by its subsequent degradation via the ubiquitin-mediated 26S proteosome pathway [8]. A 900 kDa complex, termed the I*κ*B kinase (IKK) complex, has been identified and it consists of two kinase subunits, IKK*α* and IKK*β*, and a regulatory subunit, IKK*γ* [9, 10]. These two kinase subunits form homo- or heterodimers that phosphorylate I*κ*B molecules, leading to their degradation. This activation pathway for the p50–p65 heterodimer has been referred to as the classical or canonical NF-*κ*B signaling pathway, and is dependent on the IKK*β* and IKK*γ* subunits of IKK [11]. An alternative NF-*κ*B signaling pathway has also been identified, in which IKK*α* is required and it results in the activation of the p52-RelB heterodimer [11].

One of the many mechanisms by which NF-*κ*B can be activated is by increased oxidative stress. NF-*κ*B can be activated in vitro by H_2_O_2_, and its activation can be inhibited by antioxidants, such as vitamin E or N-acetyl cysteine (NAC), or by increased expression of antioxidant enzymes [12–18]. In addition, agents that activate NF-*κ*B frequently also increase oxidative stress [19]. However, Hayakawa et al. [20] found that NAC inhibits NF-*κ*B activation independently of its antioxidant function.

NF-*κ*B has been shown to be important in the regulation of numerous genes, including many that regulate the immune response, inflammation, cell proliferation, and apoptosis [21–23]. Several inflammatory factors that are related to NF-*κ*B activation have been identified, including TNF-*α*, interleukin (IL)-6, and IL-1*β* [23, 24]. Several studies have used genetically modified mice to examine the role of NF-*κ*B subunits in these functions. Knockout mice have been developed for all NF-*κ*B subunits [25–29]; in addition, knockouts for specific tissues, such as the liver, have been developed [30, 31]. Studies in which NF-*κ*B activity has been inhibited by the deletion of one of its subunits, the inhibition of its translocation, or the expression of a dominant negative form of I*κ*B have demonstrated a clear role for NF-*κ*B in inhibiting apoptosis by tumor necrosis factor-*α* (TNF-*α*) or other apoptosis inducers in several cell types [25, 26, 32–36]. The deletion of the p65/relA subunit leads to embryonic lethality at 15-16 days of gestation, due to hepatocyte apoptosis [26]. The deletion of the p50 subunit leads to defects in the immune response involving B cells [25]. Hepatocyte apoptosis is higher in p50 −/− mice [37, 38], but it is not lethal as in the p65 knockout. However, DNA synthesis and liver regeneration were not affected by the absence of the p50 subunit following partial hepatectomy or carbon tetrachloride treatment; increased levels of p65 may have compensated for the lack of p50 [39]. Similarly, the hepatic-specific expression of a truncated I*κ*B*α* superrepressor did not affect DNA synthesis, apoptosis, or liver regeneration following partial hepatectomy, but led to increased apoptosis after treatment with TNF-*α* [40]. Also, the hepatic inflammatory response after ischemia/reperfusion was not altered in p50 −/− mice [41]. The deletion of p52 led to defects in humoral immunity and splenic architecture [28]. In RelB −/− mice, multiorgan inflammation, impaired cellular immunity, and hematopoietic abnormalities were observed [29]. The deletion of c-rel led to defects in humoral immunity and in the proliferation of T cells in response to mitogens [27]. In addition, B cells lacking p50, RelB, or c-Rel (but not p52 or p65) exhibited decreased proliferation in response to lipopolysaccharide (LPS) [25, 27, 42–44].

## 3. HEPATIC ACTIVATION OF NF-*κ*B BY PEROXISOME PROLIFERATORS

Our initial study examined whether peroxisome proliferators could activate NF-*κ*B in the liver (Table 1) [45]. Rats were fed a diet containing 0.01% ciprofibrate; control rats received the same diet without ciprofibrate. Animals were sacrificed 3, 6, or 10 days after starting treatment. NF-*κ*B DNA binding activity was monitored using electrophoretic mobility shift assays (EMSAs) with a radiolabeled NF-*κ*B probe. Low levels of NF-*κ*B were found in the liver nuclear extracts from control rats and remained unchanged over the 10-day period. Three days after the initiation of treatment, an increase in nuclear NF-*κ*B DNA binding activity was observed in treated versus control rats. NF-*κ*B levels continued to increase at six and ten days after treatment. Quantitative radioanalytic image analysis indicated that the level of induction was nearly two-fold by day 3 and increased to 4-fold by day 10. Hepatocyte nuclear factor 3 (HNF-3; foxa) is composed of a family of liver-enriched transcription factors that regulate the expression of many liver genes [46]. EMSAs with a radiolabeled HNF-3 binding motif derived from the rat transthyretin promoter showed that HNF-3 binding activity remained unchanged over the 10-day period in both treated and control rats. This indicates that ciprofibrate does not lead to a global, but rather a more restricted increase in hepatic transcription factor activity.

Following this initial observation, several additional studies have demonstrated that peroxisome proliferators activate NF-*κ*B in the livers of rats and mice, species that are sensitive to the carcinogenic effects of peroxisome proliferators (Table 1). Ciprofibrate has been found to increase the DNA binding activity of NF-*κ*B in both rats and mice [16, 17, 38, 57, 58]. Wy-14,643 and dicamba increased the DNA binding activity of NF-*κ*B in rats and/or mice, while gemfibrozil and dibutyl phthalate activated NF-*κ*B to a lesser extent in rats and not at all timepoints [49, 53, 54, 56]. Delerive et al. [52] observed that hepatic I*κ*B*α* expression was increased by ciprofibrate in mice; this finding is somewhat difficult to interpret since I*κ*B is an NF-*κ*B-regulated gene but it is also associated with inhibiting NF-*κ*B signaling. Nanji et al. [55] observed that clofibrate treatment did not affect the DNA binding activity of NF-*κ*B (this study also observed that clofibrate decreased ethanol-induced NF-*κ*B activation); however, this finding is also difficult to interpret since all rats were fed fish oil, which itself is a peroxisome proliferator [59]. All of the above studies examined NF-*κ*B activation 3 or more days after beginning peroxisome proliferator administration. In short-term studies, Rusyn et al. observed that hepatic NF-*κ*B DNA binding activity was increased shortly after a single dose of Wy-14,643, and that the increase was primarily due to increased DNA binding activity in Kupffer cells and to the presence of NADPH oxidase in Kupffer cells [50, 51]. However, after a single dose of BR-931 [47] or nafenopin [48], the DNA binding activity of NF-*κ*B in liver was not increased after 0.5–24 hours following exposure. Taken together, these data suggest that the early activation of hepatic NF-*κ*B occurs in Kupffer cells, while the activation in hepatocytes does not appear until 3 days or later after the beginning of peroxisome proliferator administration. Finally, the presence of PPAR*α* is necessary for these changes in NF-*κ*B activation to occur, since neither Wy-14,643 nor ciprofibrate affected NF-*κ*B activation in PPAR*α*-deficient mice [52, 56].

The activation of hepatic NF-*κ*B had also been examined in vitro. EMSAs demonstrated NF-*κ*B induction by ciprofibrate in peroxisome proliferator-responsive H4IIEC3 rat hepatoma cells but not in peroxisome proliferator-insensitive HepG2 human hepatoma cell lines [18]. In addition, stably transfected NF-*κ*B-regulated reporter genes were activated by ciprofibrate in H4IIEC3 cells. These changes were observed after 72 hours of exposure, with the increase in fatty acyl CoA oxidase activity being observed at 24 hours, the first time point tested. This reporter gene activation was blocked by the antioxidants *N*-acetylcysteine and vitamin E. West et al. [60] examined the activation of NF-*κ*B in cultured mouse hepatocytes in response to nafenopin. After a 4-hour exposure, the DNA binding activity of NF-*κ*B was increased. Using human HuH7 hepatoma cells, Kleemann et al. [61] found that Wy-14,643 increased I*κ*B*α* protein levels and decreased the nuclear translocation of NF-*κ*B. In addition, the peroxisome proliferators Wy-14,643 and fenofibrate decreased interleukin- (IL-) 1*β*-induced C-reactive protein expression. Delerive et al. [52] also found that I*κ*B*α* expression and protein levels were increased by Wy-14,643 in primary human hepatocytes and that IL-1*β*-induced cyclooxygenase- (COX-) 2 protein levels were decreased by Wy-14,643. These studies suggest that rodent hepatic NF-*κ*B activation is due to, at least in part, activation in hepatocytes. In human liver cells and cell lines, however, NF-*κ*B activation is not affected or is decreased by peroxisome proliferator administration.

## 4. IMPORTANCE OF NF-*κ*B IN HEPATOCARCINOGENESIS BY PEROXISOME PROLIFERATORS

An important question is whether NF-*κ*B activation by peroxisome proliferators is necessary for carcinogenesis by peroxisome proliferators, as well as the induction of changes in cell proliferation, apoptosis, and gene expression. If NF-*κ*B activation does contribute to the promoting activity of peroxisome proliferators, one would predict that if the activity of NF-*κ*B were diminished, the enhancement of cell proliferation and carcinogenesis as well as the inhibition of apoptosis by peroxisome proliferators would be decreased. Several studies have examined this question, using mice in which the p50 subunit of NF-*κ*B has been deleted. In the first study, the effect of p50 deletion on cell proliferation, apoptosis, and related gene expression was examined [38]. Wild-type and p50 −/− mice were fed a diet with or without 0.01% ciprofibrate for 10 days. NF-*κ*B DNA binding activity was present and increased after ciprofibrate treatment in wild-type mice, but was not detected in p50 −/− mice. The untreated p50 −/− mice had a higher level of hepatic cell proliferation, as measured by BrdU labeling, than did untreated wild-type mice. However, the increase in proliferation was greater in ciprofibrate-fed wild-type mice than in ciprofibrate-fed p50 −/− mice. The apoptotic index was low in wild-type mice in the presence or absence of ciprofibrate. Apoptosis was increased in untreated p50 −/− mice compared to wild-type mice; apoptosis was reduced in p50 −/− mice after ciprofibrate feeding. Because increased cell proliferation in the liver is associated with increased activator protein-1 (AP-1) activity, the expression of genes in the Fos and Jun families of transcription factors was examined. The c-Jun and JunB mRNA levels were higher in untreated p50 −/− mice than in untreated wild-type mice; c-Jun mRNA levels increased whereas JunB mRNA levels decreased in both groups after ciprofibrate treatment. However, c-Jun and JunB protein levels were the same in untreated wild-type and p50 −/− mice, and increased in both groups after ciprofibrate treatment. Apoptosis-related gene expression was also examined, and several apoptosis-related mRNAs were higher in untreated p50 −/− mice compared to untreated wild-type mice; expression of these genes increased in both groups after ciprofibrate treatment. These data indicate that NF-*κ*B contributes to the proliferative and apoptotic changes that occur in the liver in response to ciprofibrate.

The role of NF-*κ*B in the inhibition of apoptosis by peroxisome proliferators has also been examined in vitro. Using primary rat hepatocytes, Cosulich et al. [62] infected cells with an adenovirus containing a dominant negative form of IKK2. The dominant negative IKK2 induced apoptosis in the hepatocytes, which could not be inhibited by the addition of nafenopin. These data indicate that NF-*κ*B activation is essential for the inhibition of apoptosis by peroxisome proliferators.

The role of NF-*κ*B in the promotion of hepatocarcinogenesis by Wy-14,643 has been examined, using p50 −/− mice [63]. The p50 −/− and wild-type mice were first administered diethylnitrosamine (DEN) as an initiating agent. Mice were then fed a control diet or a diet containing 0.05% Wy-14,643 for 38 weeks. As expected, wild-type mice receiving DEN only developed a low incidence of tumors, and the majority of wild-type mice receiving both DEN and Wy-14,643 developed tumors. However, no tumors were seen in any of the p50 −/− mice. Treatment with DEN/Wy-14,643 increased both cell proliferation and apoptosis in wild-type and p50 −/− mice; DEN treatment alone had no effect. In the DEN/Wy-14,643-treated mice, cell proliferation and apoptosis were slightly lower in the p50 −/− mice than in the wild-type mice. These data demonstrate that NF-*κ*B is involved in the promotion of hepatic tumors by the peroxisome proliferator Wy-14,643; however, in this study, the difference in tumor incidence could not be attributed to alterations in either cell proliferation or apoptosis.

## 5. MECHANISMS BY WHICH PEROXISOME PROLIFERATORS INFLUENCE NF-*κ*B ACTIVATION

The studies discussed above showed that peroxisome proliferators activate hepatic NF-*κ*B, except possibly for very short exposure periods, and that NF-*κ*B activation is necessary for the promoting activity and associated biochemical activities of peroxisome proliferators. The mechanisms by which peroxisome proliferators activate NF-*κ*B have been examined in several studies. These studies can be divided into two main groups: (1) those taking place in nonhepatic cells or nonrodent hepatocytes, or in which the exposure time was short; and (2) those in liver, in which the exposure time was longer, usually greater than one week. The former studies involved alterations in NF-*κ*B in the absence of changes in gene expression brought about by PPAR*α* activation in rodent liver. For the longer studies, however, changes in gene expression and cell metabolism in response to PPAR*α* activation have occurred. These include the induction of the peroxisomal *β*-oxidation pathway including fatty acyl CoA oxidase (FAO) and the cytochrome P-450 4A (CYP4A) family. FAO produces hydrogen peroxide as a by-product, and CYP4A may also produce reactive oxygen species. PPAR*α* activation also results in a decrease in the activities of cellular antioxidant enzymes such as glutathione peroxidase, glutathione S-transferase, and DT-diaphorase, and in the concentrations of cellular antioxidants such as vitamin E [7]. Therefore, oxidative stress may be an important mechanism in the activation of NF-*κ*B by peroxisome proliferators.

In tissues that are not responsive to the peroxisome proliferative and carcinogenic effects to peroxisome proliferators, such as human hepatocytes and nonhepatocyte tissues and cells, the administration of PPAR*α* activators clearly leads to a decrease in NF-*κ*B activation and NF-*κ*B-regulated gene expression. These include kidney cells in vitro [64], human aortic smooth muscle cells [52, 65], human HuH7 hepatoma cells [61], primary human hepatocytes [52], human endothelial cells [66], Cos-1 cells [67], and mouse splenocytes in vivo [68]. In these cases, PPAR*α* decreased NF-*κ*B activation by the direct interaction with p65 [65] and/or by increasing I*κ*B*α* expression [52]. The administration of peroxisome proliferators also decreased the expression and/or protein levels of NF-*κ*B-regulated inflammatory genes, including IL-6 [65, 68], IL-12 [68], C-reactive protein [61], vascular cell adhesion molecule-1 (VCAM-1) [66], and COX-2 [67]. On the other hand, inhibition of the NF-*κ*B signaling pathway by inactivating the NF-*κ*B essential modulator (NEMO) gene in rodent liver leads to a decrease in the expression of PPAR*α* [69].

Several lines of evidence support the hypothesis that NF-*κ*B activation after one week or more of exposure to peroxisome proliferators is mediated by oxidative stress produced by peroxisome proliferators. First, overexpression of the hydrogen peroxide-producing enzyme that is induced by peroxisome proliferators, FAO, is sufficient to activate NF-*κ*B in Cos-1 cells [70]. In addition, FAO overexpression in Cos-1 cells, in the presence of an H_2_O_2_-generating substrate, can activate an NF-*κ*B-regulated reporter gene. Electrophoretic mobility shift assays further demonstrated that FAO expression increases nuclear NF-*κ*B DNA binding activity in a dose-dependent manner. The antioxidants vitamin E and catalase can inhibit this activation [70].

Second, overexpression of the hydrogen peroxide-detoxifying enzyme catalase in the livers of transgenic mice inhibits the activation of NF-*κ*B by ciprofibrate [16]. In this study, mice overexpressing catalase in the liver or non-transgenic littermates were fed either 0.01% ciprofibrate or a control diet for 21 days. FAO activity was not significantly affected by catalase overexpression although the ratio of FAO to catalase was significantly decreased in transgenic animals. Ciprofibrate increased NF-*κ*B DNA binding activity in the livers of non-transgenic mice, but this increase was inhibited by catalase overexpression. In addition, the ciprofibrate-induced increase in hepatocyte proliferation was decreased by catalase overexpression, indicating a possible role for NF-*κ*B in cell proliferation by peroxisome proliferators.

Third, studies in species with different responses to peroxisome proliferators support a role for oxidative stress in NF-*κ*B activation. Rats and mice are sensitive to the hepatocarcinogenic and cell proliferation-inducing effects of peroxisome proliferators whereas other species, such as Syrian hamsters, are not [71, 72]. Therefore, we examined the effects of three different peroxisome proliferators on antioxidant enzymes, antioxidant vitamins, and NF-*κ*B activation in rats and Syrian hamsters [53, 73, 74]. The peroxisome proliferators Wy-14,643, gemfibrozil, and dibutyl phthalate were administered to animals for 6, 34, or 90 days. In rats, decreases in glutathione reductase (GR), glutathione S-transferase (GST), and selenium-dependent glutathione peroxidase (GPx) were observed following peroxisome proliferator treatment at various time points. In hamsters, a higher basal level of activities for GR, GST, and selenium GPx was observed as compared to rats. In addition, hamsters showed decreases in GR and GST activities following peroxisome proliferator treatment. Interestingly, selenium-GPx activity was increased in hamsters following peroxisome proliferator treatment. Treatment for 90 days with Wy-14,643 resulted in no change in GPx1 mRNA in rats and increased GPx1 mRNA in hamsters. In both rats and hamsters treated with Wy-14,643, we observed decreases in *α*-tocopherol content and total superoxide dismutase (SOD) activity. Conversely, DT-diaphorase activity was decreased following Wy-14,643 treatment in rats at all time points and doses, but only sporadically affected in hamsters. Rats and hamsters treated with DBP demonstrated increased SOD activity at 6 days; however, in the rat, DBP decreased SOD activity at 90 days and *α*-tocopherol content was decreased throughout. In gemfibrozil-treated rats and hamsters, a decrease in *α*-tocopherol content and an increase in DT-diaphorase activity were observed. In either species, no consistent trend was observed in total ascorbic acid content after treatment with any of the peroxisome proliferators. NF-*κ*B activation was evaluated by EMSA. Wy-14,643 increased the DNA binding activity of NF-*κ*B at all three timepoints in rats and produced the highest activation of the three chemicals tested (Table 1). Gemfibrozil and DBP increased NF-*κ*B activation to a less extent in rats and not at all times. There were no differences in hepatic NF-*κ*B levels between control hamsters and hamsters treated with any of the peroxisome proliferators. These studies show that NF-*κ*B is not activated by peroxisome proliferators in hamsters, which have much higher levels of the antioxidant enzymes glutathione peroxidase, glutathione S-transferase, glutathione reductase, and DT-diaphorase, and which are not responsive to the carcinogenic effects of the peroxisome proliferators.

Finally, the antioxidant, vitamin E, inhibits ciprofibrate-induced NF-*κ*B activation, both in vivo and in vitro. In an in vitro study [18], NF-*κ*B-regulated reporter genes were stably transfected into rat hepatoma H4IIEC3 cells. The ciprofibrate-induced increase in luciferase activity after 72 hours of exposure was blocked by the addition of *α*-tocopheryl acetate. N-acetyl cysteine also inhibited the ciprofibrate-induced increase. In the in vivo study [17], thirty-six male Sprague-Dawley rats were fed a purified diet containing varying levels of vitamin E (10, 50, 250 ppm *α*-tocopheryl acetate). After 28 days, seven animals per vitamin E group received 0.01% ciprofibrate in the diet for 10 days. Increased dietary *α*-tocopherol acetate inhibited CIP-induced NF-*κ*B DNA binding. Since NF-*κ*B translocates to the nucleus upon the phosphorylation and degradation of I*κ*B, we also used western blots to measure cytosolic protein levels of I*κ*B*α*, I*κ*B*β*, and I*κ*B kinases: IKK*α* and IKK*β*. However, I*κ*B*α* protein levels were decreased in all three CIP-treated groups, with the 10 ppm vitamin E diet also decreasing I*κ*B*α* levels in control rats. No difference in I*κ*B*β* protein levels was observed among any of the groups. The CIP-treated rats generally had lower protein levels of IKK*α* and IKK*β*.

An important question is whether vitamin E is exerting some of its effects by blocking the activation of NF-*κ*B. The use of NF-*κ*B knockout models may provide answers to this question. A study has addressed this question by examining if the inhibition of NF-*κ*B by vitamin E is necessary for vitamin E's effects on the induction of cell proliferation by the peroxisome proliferator ciprofibrate and on the inhibition of apoptosis by ciprofibrate [57]. Wild-type and p50 −/− mice were administered ciprofibrate and one of two levels of vitamin E (10 or 250 mg/kg diet). Vitamin E inhibited ciprofibrate-induced cell proliferation only in the p50 −/− mice. Dietary vitamin E also increased apoptosis and increased the GSH/GSSG ratio in both wild-type and p50 −/− mice. This study suggests that vitamin E does not act by blocking NF-*κ*B activation, indicating that vitamin E is acting by other molecular mechanisms.

## 6. CONCLUSIONS

In summary, the administration of most peroxisome proliferators leads to the activation of NF-*κ*B in the liver of rats and mice. This activation appears to be necessary for the tumor-promoting activity and for the induction of cell proliferation by peroxisome proliferators. The activation of NF-*κ*B appears to be mediated at least in part by the induction of oxidative stress by peroxisome proliferators. Future studies examining the mechanisms by which NF-*κ*B is altered by PPAR*α* activation will need to clearly distinguish between those changes brought about directly by PPAR*α* and those brought about as a result of changes in gene expression through PPAR*α* (such as the peroxisomal *β*-oxidation pathway). The identity of specific NF-*κ*B-regulated genes after the administration of peroxisome proliferators, particularly genes related to cell proliferation and apoptosis, will also need to be determined in future studies.

## Figures and Tables

**Table 1:** Effect of peroxisome proliferators on the activation of NF-*κ*B in the liver in vivo.

Authors	Species	Agent	Dose	Time Points	Endpoint	Effect
Li et al., 1996 [45]	Rats	Ciprofibrate	0.01% in diet	3, 6, 10 days	EMSA	Increased
Ohmura et al., 1996 [47]	Rats	BR-931	250 mg/kg single p.o. dose	0.5–5 hr after single dose	EMSA	No effect
Menegazzi et al., 1997 [48]	Rats	Nafenopin	200 mg/kg single p.o. dose	0.5–24 hr after single dose	EMSA	No effect
Nilakantan et al., 1998 [16]	Mice	Ciprofibrate	0.01% in diet	21 days	EMSA	Increased
Espandiari et al., 1998 [49]	Rats	Dicamba	1 or 3% in diet	7 days	EMSA	Increased
Rusyn et al., 1998 [50]	Rats	Wy-14,643	100 mg/kg single p.o. dose	1–36 hr after single dose	EMSA	Increased at 2 and 8 hr; no change at 1, 24, and 36 hr
Rusyn et al., 2000 [51]	Rats	Wy-14,643	100 mg/kg single p.o. dose	2 hr after single dose	EMSA	Increased
	Mice	Wy-14,643	100 mg/kg single p.o. dose	2–24 hr after single dose	EMSA	Increased
Delerive et al., 2000 [52]	Mice	Ciprofibrate	0.05% in diet	2 weeks	I*κ*B*α* expression	Increased in wild-type but not PPAR*α* −/− mice
Tharappel et al., 2001 [53]	Rats	Wy-14,643	0.05 or 0.005% in diet	6, 34, 90 days	EMSA	Increased
		Gemfibrozil	0.1 or 1.6% in diet	6, 34 days	EMSA	No effect
		Gemfibrozil	0.1 or 1.6% in diet	90 days	EMSA	Increased only at lower dose
		Dibutyl phthalate	0.5 or 2.0% in diet	6 days	EMSA	No effect
		Dibutyl phthalate	0.5 or 2.0% in diet	34, 90 days	EMSA	Increased
	Hamsters	Wy-14,643	0.05 or 0.005% in diet	6, 34, 90 days	EMSA	No effect
		Gemfibrozil	0.6 or 2.4% in diet	6, 34, 90 days	EMSA	No effect
		Dibutyl phthalate	0.5 or 2.0% in diet	6, 34, 90 days	EMSA	No effect
Fischer et al., 2002 [54]	Rats	Wy-14,643	0.1% in diet	10 days	EMSA	Increased
Tharappel et al., 2003 [38]	Mice	Ciprofibrate	0.01% in diet	10 days	EMSA	Increased
Calfee-Mason et al., 2004 [17]	Rats	Ciprofibrate	0.01% in diet	10 days	EMSA	Increased
Nanji et al., 2004 [55]	Rats	Clofibrate	100 mg/kg p.o. daily + fish oil	4 weeks	EMSA	No effect compared to fish oil alone; decreased ethanol-induced activation
Woods et al., 2007 [56]	Mice	Wy-14,643	0.1% in diet	1 week, 5 weeks, 5 months	EMSA	Increased in wild-type and NADPH oxidase-deficient mice; no effect in PPAR*α*-deficient mice
Calfee-Mason et al., 2008 [57]	Mice	Ciprofibrate	0.01% in diet	10 days	EMSA	Increased

## References

[1] Rao MS, Reddy JK (1987). Peroxisome proliferation and hepatocarcinogenesis. *Carcinogenesis*.

[2] Cattley RC, DeLuca J, Elcombe C (1998). Do peroxisome proliferating compounds pose a hepatocarcinogenic hazard to humans?. *Regulatory Toxicology and Pharmacology*.

[3] Peters JM, Cattley RC, Gonzalez FJ (1997). Role of PPAR*α* in the mechanism of action of the nongenotoxic carcinogen and peroxisome proliferator Wy-14,643. *Carcinogenesis*.

[4] Schoonjans K, Staels B, Auwerx J (1996). Role of the peroxisome proliferator-activated receptor (PPAR) in mediating the effects of fibrates and fatty acids on gene expression. *Journal of Lipid Research*.

[5] Eacho PI, Feller DR, Witiak DT, Newman HAI, Feller DR (1991). Hepatic peroxisome proliferation induced by hypolipidemic drugs and other chemicals. *Antilipidemic Drugs, Medicinal, Chemical and Biochemical Aspects*.

[6] Lock EA, Mitchell AM, Elcombe CR (1989). Biochemical mechanisms of induction of hepatic peroxisome proliferation. *Annual Review of Pharmacology and Toxicology*.

[7] O'Brien ML, Spear BT, Glauert HP (2005). Role of oxidative stress in peroxisome proliferator-mediated carcinogenesis. *Critical Reviews in Toxicology*.

[8] Karin M, Lin A (2002). NF-*κ*B at the crossroads of life and death. *Nature Immunology*.

[9] Karin M, Delhase M (2000). The I*κ*B kinase (IKK) and NF-*κ*B: key elements of proinflammatory signalling. *Seminars in Immunology*.

[10] Zandi E, Rothwarf DM, Delhase M, Hayakawa M, Karin M (1997). The I*κ*B kinase complex (IKK) contains two kinase subunits, IKK*α* and IKK*β*, necessary for I*κ*B phosphorylation and NF-*κ*B activation. *Cell*.

[11] Karin M (2006). NF-*κ*B and cancer: mechanisms and targets. *Molecular Carcinogenesis*.

[12] Staal FJT, Roederer M, Herzenberg LA, Herzenberg LA (1990). Intracellular thiols regulate activation of nuclear factor *κ*B and transcription of human immunodeficiency virus. *Proceedings of the National Academy of Sciences of the United States of America*.

[13] Schreck R, Rieber P, Baeuerle PA (1991). Reactive oxygen intermediates as apparently widely used messengers in the activation of the NF-*κ*B transcription factor and HIV-1. *The EMBO Journal*.

[14] Schreck R, Albermann K, Baeuerle PA (1992). Nuclear factor *κ*B: an oxidative stress-responsive transcription factor of eukaryotic cells (a review). *Free Radical Research Communications*.

[15] Meyer M, Schreck R, Baeuerle PA (1993). H_2_O_2_ and antioxidants have opposite effects on activation of NF-*κ*B and AP-1 in intact cells: AP-1 as secondary antioxidant-responsive factor. *The EMBO Journal*.

[16] Nilakantan V, Spear BT, Glauert HP (1998). Liver-specific catalase expression in transgenic mice inhibits NF-*κ*B activation and DNA synthesis induced by the peroxisome proliferator ciprofibrate. *Carcinogenesis*.

[17] Calfee-Mason KG, Spear BT, Glauert HP (2004). Effects of vitamin E on the NF-*κ*B pathway in rats treated with the peroxisome proliferator, ciprofibrate. *Toxicology and Applied Pharmacology*.

[18] Li Y, Glauert HP, Spear BT (2000). Activation of nuclear factor-*κ*B by the peroxisome proliferator ciprofibrate in H4IIEC3 rat hepatoma cells and its inhibition by the antioxidants *N*-acetylcysteine and vitamin E. *Biochemical Pharmacology*.

[19] Schmidt KN, Amstad P, Cerutti P, Baeuerle PA (1995). The roles of hydrogen peroxide and superoxide as messengers in the activation of transcription factor NF-*κ*B. *Chemistry & Biology*.

[20] Hayakawa M, Miyashita H, Sakamoto I (2003). Evidence that reactive oxygen species do not mediate NF-*κ*B activation. *The EMBO Journal*.

[21] FitzGerald MJ, Webber EM, Donovan JR, Fausto N (1995). Rapid DNA binding by nuclear factor *κ*B in hepatocytes at the start of liver regeneration. *Cell Growth & Differentiation*.

[22] Beg AA, Sha WC, Bronson RT, Ghosh S, Baltimore D (1995). Embryonic lethality and liver degeneration in mice lacking the RelA component of NF-*κ*B. *Nature*.

[23] Vanden Berghe W, Vermeulen L, Delerive P, De Bosscher K, Staels B, Haegeman G (2003). A paradigm for gene regulation: inflammation, NF-*κ*B and PPAR. *Advances in Experimental Medicine and Biology*.

[24] Maeda S, Omata M (2008). Inflammation and cancer: role of nuclear factor-kappaB activation. *Cancer Science*.

[25] Sha WC, Liou H-C, Tuomanen EI, Baltimore D (1995). Targeted disruption of the p50 subunit of NF-*κ*B leads to multifocal defects in immune responses. *Cell*.

[26] Beg AA, Sha WC, Bronson RT, Ghosh S, Baltimore D (1995). Embryonic lethality and liver degeneration in mice lacking the RelA component of NF-*κ*B. *Nature*.

[27] Kontgen F, Grumont RJ, Strasser A (1995). Mice lacking the c-rel proto-oncogene exhibit defects in lymphocyte proliferation, humoral immunity, and interleukin-2 expression. *Genes & Development*.

[28] Franzoso G, Carlson L, Poljak L (1998). Mice deficient in nuclear factor (NF)-*κ*B/p52 present with defects in humoral responses, germinal center reactions, and splenic microarchitecture. *Journal of Experimental Medicine*.

[29] Weih F, Carrasco D, Durham SK (1995). Multiorgan inflammation and hematopoietic abnormalities in mice with a targeted disruption of RelB, a member of the NF-*κ*B/Rel family. *Cell*.

[30] Pikarsky E, Porat RM, Stein I (2004). NF-*κ*B functions as a tumour promoter in inflammation-associated cancer. *Nature*.

[31] Sakurai T, Maeda S, Chang L, Karin M (2006). Loss of hepatic NF-*κ*B activity enhances chemical hepatocarcinogenesis through sustained c-Jun N-terminal kinase 1 activation. *Proceedings of the National Academy of Sciences of the United States of America*.

[32] Van Antwerp DJ, Martin SJ, Kafri T, Green DR, Verma IM (1996). Suppression of TNF-*α*-induced apoptosis by NF-*κ*B. *Science*.

[33] Wang C-Y, Mayo MW, Baldwin AS (1996). TNF- and cancer therapy-induced apoptosis: potentiation by inhibition of NF-*κ*B. *Science*.

[34] Beg AA, Baltimore D (1996). An essential role for NF-*κ*B in preventing TNF-*α*-induced cell death. *Science*.

[35] Schoemaker MH, Ros JE, Homan M (2002). Cytokine regulation of pro- and anti-apoptotic genes in rat hepatocytes: NF-*κ*B-regulated inhibitor of apoptosis protein 2 (cIAP2) prevents apoptosis. *Journal of Hepatology*.

[36] Xu Y, Bialik S, Jones BE (1998). NF-*κ*B inactivation converts a hepatocyte cell line TNF-*α* response from proliferation to apoptosis. *American Journal of Physiology*.

[37] Lu Z, Lee EY, Robertson LW, Glauert HP, Spear BT (2004). Effect of 2,2′,4,4′,5,5′-hexachlorobiphenyl (PCB-153) on hepatocyte proliferation and apoptosis in mice deficient in the p50 subunit of the transcription factor NF-*κ*B. *Toxicological Sciences*.

[38] Tharappel JC, Nalca A, Owens AB (2003). Cell proliferation and apoptosis are altered in mice deficient in the NF-*κ*B p50 subunit after treatment with the peroxisome proliferator ciprofibrate. *Toxicological Sciences*.

[39] DeAngelis RA, Kovalovich K, Cressman DE, Taub R (2001). Normal liver regeneration in p50/nuclear factor *κ*B1 knockout mice. *Hepatology*.

[40] Chaisson ML, Brooling JT, Ladiges W, Tsai S, Fausto N (2002). Hepatocyte-specific inhibition of NF-*κ*B leads to apoptosis after TNF treatment, but not after partial hepatectomy. *Journal of Clinical Investigation*.

[41] Kato A, Edwards MJ, Lentsch AB (2002). Gene deletion of NF-*κ*B p50 does not alter the hepatic inflammatory response to ischemia/reperfusion. *Journal of Hepatology*.

[42] Horwitz BH, Zelazowski P, Shen Y (1999). The p65 subunit of NF-*κ*B is redundant with p50 during B cell proliferative responses, and is required for germline C_H_ transcription and class switching to IgG3. *The Journal of Immunology*.

[43] Snapper CM, Zelazowski P, Rosas FR (1996). B cells from p50/NF-*κ*B knockout mice have selective defects in proliferation, differentiation, germ-line CH transcription, and Ig class switching. *The Journal of Immunology*.

[44] Snapper CM, Rosas FR, Zelazowski P (1996). B cells lacking RelB are defective in proliferative responses, but undergo normal B cell maturation to Ig secretion and Ig class switching. *Journal of Experimental Medicine*.

[45] Li Y, Leung LK, Glauert HP, Spear BT (1996). Treatment of rats with the peroxisome proliferator ciprofibrate results in increased liver NF-*κ*B activity. *Carcinogenesis*.

[46] Costa RH, Tronche R, Yaniv M (1994). HNF3 protein family. *Liver Gene Expression*.

[47] Ohmura T, Ledda-Columbano GM, Piga R (1996). Hepatocyte proliferation induced by a single dose of a peroxisome proliferator. *American Journal of Pathology*.

[48] Menegazzi M, Carcereri-De Prati A, Suzuki H (1997). Liver cell proliferation induced by nafenopin and cyproterone acetate is not associated with increases in activation of transcription factors NF-*κ*B and AP-1 or with expression of tumor necrosis factor *α*. *Hepatology*.

[49] Espandiari P, Ludewig G, Glauert HP, Robertson LW (1998). Activation of hepatic NF-*κ*B by the herbicide Dicamba (2-methoxy-3,6-dichlorobenzoic acid) in female and male rats. *Journal of Biochemical and Molecular Toxicology*.

[50] Rusyn I, Tsukamoto H, Thurman RG (1998). WY-14 643 rapidly activates nuclear factor *κ*B in Kupffer cells before hepatocytes. *Carcinogenesis*.

[51] Rusyn I, Yamashina S, Segal BH (2000). Oxidants from nicotinamide adenine dinucleotide phosphate oxidase are involved in triggering cell proliferation in the liver due to peroxisome proliferators. *Cancer Research*.

[52] Delerive P, Gervois P, Fruchart J-C, Staels B (2000). Induction of I*κ*B*α* expression as a mechanism contributing to the anti-inflammatory activities of peroxisome proliferator-activated receptor-*α* activators. *The Journal of Biological Chemistry*.

[53] Tharappel JC, Cunningham ML, Spear BT, Glauert HP (2001). Differential activation of hepatic NF-*κ*B in rats and hamsters by the peroxisome proliferators Wy-14,643, gemfibrozil, and dibutyl phthalate. *Toxicological Sciences*.

[54] Fischer JG, Glauert HP, Yin T, Sweeney-Reeves ML, Larmonier N, Black MC (2002). Moderate iron overload enhances lipid peroxidation in livers of rats, but does not affect NF-*κ*B activation induced by the peroxisome proliferator, Wy-14,643. *The Journal of Nutrition*.

[55] Nanji AA, Dannenberg AJ, Jokelainen K, Bass NM (2004). Alcoholic liver injury in the rat is associated with reduced expression of peroxisome proliferator-*α* (PPAR*α*)-regulated genes and is ameliorated by PPAR*α* activation. *Journal of Pharmacology and Experimental Therapeutics*.

[56] Woods CG, Burns AM, Bradford BU (2007). WY-14,643-induced cell proliferation and oxidative stress in mouse liver are independent of NADPH oxidase. *Toxicological Sciences*.

[57] Calfee-Mason KG, Lee EY, Spear BT, Glauert HP (2008). Role of the p50 subunit of NF-*κ*B in vitamin E-induced changes in mice treated with the peroxisome proliferator, ciprofibrate. *Food and Chemical Toxicology*.

[58] Li Y, Glauert HP, Spear BT (1996). NF*κ*B activity is increased by the peroxisome proliferator ciprofibrate in rats. *Proceedings of the American Association of Cancer Research*.

[59] Neschen S, Moore I, Regittnig W (2002). Contrasting effects of fish oil and safflower oil on hepatic peroxisomal and tissue lipid content. *American Journal of Physiology*.

[60] West DA, James NH, Cosulich SC (1999). Role for tumor necrosis factor *α* receptor 1 and interleukin-1 receptor in the suppression of mouse hepatocyte apoptosis by the peroxisome proliferator nafenopin. *Hepatology*.

[61] Kleemann R, Verschuren L, de Rooij B-J (2004). Evidence for anti-inflammatory activity of statins and PPAR*α* activators in human C-reactive protein transgenic mice in vivo and in cultured human hepatocytes in vitro. *Blood*.

[62] Cosulich SC, James NH, Needham MRC, Newham PP, Bundell KR, Roberts RA (2000). A dominant negative form of IKK2 prevents suppression of apoptosis by the peroxisome proliferator nafenopin. *Carcinogenesis*.

[63] Glauert HP, Eyigor A, Tharappel JC, Cooper S, Lee EY, Spear BT (2006). Inhibition of hepatocarcinogenesis by the deletion of the p50 subunit of NF-*κ*B in mice administered the peroxisome proliferator Wy-14,643. *Toxicological Sciences*.

[64] Inoue I, Itoh F, Aoyagi S (2002). Fibrate and statin synergistically increase the transcriptional activities of PPAR*α*/RXR*α* and decrease the transactivation of NF*κ*B. *Biochemical and Biophysical Research Communications*.

[65] Delerive P, De Bosscher K, Besnard S (1999). Peroxisome proliferator-activated receptor *α* negatively regulates the vascular inflammatory gene response by negative cross-talk with transcription factors NF-*κ*B and AP-1. *The Journal of Biological Chemistry*.

[66] Marx N, Sukhova GK, Collins T, Libby P, Plutzky J (1999). PPAR*α* activators inhibit cytokine-induced vascular cell adhesion molecule-1 expression in human endothelial cells. *Circulation*.

[67] Staels B, Koenig W, Habib A (1998). Activation of human aortic smooth-muscle cells is inhibited by PPAR*α* but not by PPAR*γ* activators. *Nature*.

[68] Poynter ME, Daynes RA (1998). Peroxisome proliferator-activated receptor *α* activation modulates cellular redox status, represses nuclear factor-*κ*B signaling, and reduces inflammatory cytokine production in aging. *The Journal of Biological Chemistry*.

[69] Wunderlich FT, Luedde T, Singer S (2008). Hepatic NF-*κ*B essential modulator deficiency prevents obesity-induced insulin resistance but synergizes with high-fat feeding in tumorigenesis. *Proceedings of the National Academy of Sciences of the United States of America*.

[70] Li Y, Tharappel JC, Cooper S, Glenn M, Glauert HP, Spear BT (2000). Expression of the hydrogen peroxide-generating enzyme fatty acyl CoA oxidase activates NF-*κ*B. *DNA and Cell Biology*.

[71] Lake BG, Evans JG, Cunninghame ME, Price RJ (1993). Comparison of the hepatic effects of nafenopin and WY-14,643 on peroxisome proliferation and cell replication in the rat and Syrian hamster. *Environmental Health Perspectives*.

[72] Durnford JM, Hejtmancik MR, Kurtz PJ (1998). Peroxisomal enzyme activity and cell proliferation in rats, mice and hamsters exposed for 13-weeks to Wy-14,643 and gemfibrozil. *Toxicological Sciences*.

[73] O'Brien ML, Cunningham ML, Spear BT, Glauert HP (2001). Effects of peroxisome proliferators on glutathione and glutathione-related enzymes in rats and hamsters. *Toxicology and Applied Pharmacology*.

[74] O'Brien ML, Twaroski TP, Cunningham ML, Glauert HP, Spear BT (2001). Effects of peroxisome proliferators on antioxidant enzymes and antioxidant vitamins in rats and hamsters. *Toxicological Sciences*.

